# Stroke‐Like Migraine Attacks After Radiation Therapy (SMART) Syndrome: A Case Report and Review of a Rare Delayed Complication of Cranial Irradiation

**DOI:** 10.1002/ccr3.71952

**Published:** 2026-01-28

**Authors:** Shiv Jha, Yugant Khand, Surabhi Ranjan, Monika Pandit, Eric Mariuma

**Affiliations:** ^1^ Department of Neurology, Montefiore Medical Center Albert Einstein College of Medicine Bronx New York USA; ^2^ Nepalese Army Institute of Health Sciences ‐ College of Medicine Kathmandu Nepal; ^3^ Department of Neurology, Cleveland Clinic Florida Weston Florida USA; ^4^ Department of Internal Medicine, Jacobi Medical Center/North Central Bronx Hospital Bronx New York USA

**Keywords:** astrocytoma, case report, cranial radiation, delayed radiation effects, SMART syndrome, stroke mimics, stroke‐like migraine

## Abstract

SMART syndrome is a rare, delayed complication of cranial irradiation that can mimic stroke, tumor recurrence, or autoimmune encephalitis. Early recognition based on clinical‐radiologic features is critical to avoid misdiagnosis and unnecessary interventions since conservative management often leads to complete recovery.

## 1 Introduction

Stroke‐like migraine attacks after radiation therapy (SMART) syndrome is a rare, delayed, and reversible neurological complication of cranial irradiation [1]. The syndrome occurs few years to decades after exposure to radiation with symptoms of transient stroke‐like neurological deficits accompanied by migraine‐type headaches. It can present in any age group who have undergone radiotherapy for various intracranial pathologies. While the exact pathophysiological mechanism is not elucidated, SMART is believed to be due to a complex interplay of radiation‐induced damage to brain vasculature and neuronal tissues [2]. The neurological dysfunction is due to the transient failure of cerebrovascular autoregulation, which results in transient focal hyperperfusion and vasogenic edema. The low threshold for cortical spreading depression due to radiation can cause trigemino‐vascular activation causing migraines. The irradiated cortex can create an epileptogenic focus that can trigger seizures. Due to non‐specific presentation and overlap with other post‐radiation conditions such as tumor recurrence, lepto or pachymeningeal metastasis, autoimmune encephalitis, peri‐ictal pseudoprogression, or stroke, it is critical care to recognize and avoid misdiagnosis or unnecessary invasive interventions. Clinician and patient awareness are essential to ensure timely supportive management and appropriate long‐term follow‐up for SMART syndrome. We report a case of SMART syndrome in a patient with cranial irradiation 35 years ago following astrocytoma resection. This case presents a diagnostic challenge with a combination of transient neurological symptoms and radiographic cortical abnormalities following cranial irradiation, which can mimic tumor recurrence, subacute stroke, or autoimmune encephalitis.

## 2 Case Presentation

### 2.1 Case History

A 69‐year‐old woman with a significant medical history of peripheral neuropathy, hypothyroidism, hyperlipidemia, gastro‐esophageal reflux disease, and a past history of right parietal astrocytoma status post‐resection and radiation therapy 35 years ago presented to the emergency department on with increasing left‐sided weakness, nausea, vomiting, and urinary incontinence followed by a fall. She had a history of unsteady gait for the past 5 years and reported episodic “dead arm” in her left upper extremity over the past several months. These symptoms lasted approximately 5 min and comprised transient loss of voluntary movement without loss of consciousness, during which she would hold the affected arm with her right hand. She also reported new‐onset spontaneous motion in the left upper extremity and dragging of the left leg. The patient denied recent fever or bowel changes but reported a viral‐like gastrointestinal illness briefly one to 2 weeks prior to admission.

### 2.2 Differentials, Investigation, Treatment

**FIGURE 1:**
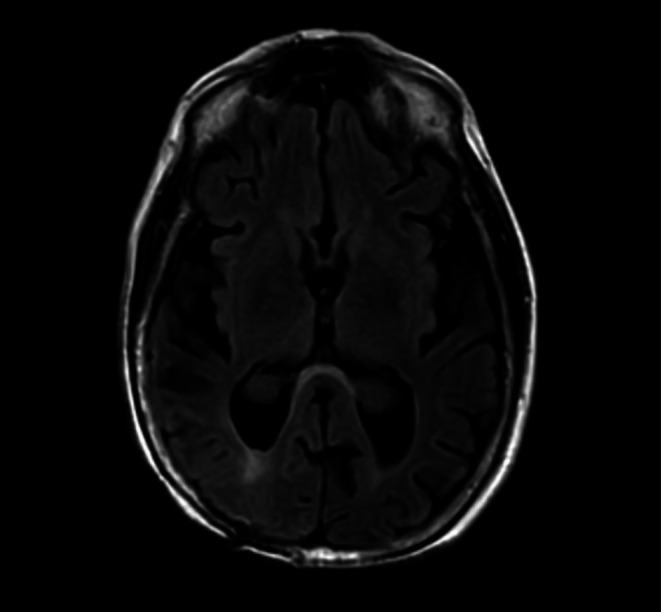
MRI brain showed a right parietal cranioplasty with a post‐surgical cavity in the paramedian right parietal lobe, gliosis, and benign choroid plexus xanthogranuloma.

Upon hospitalization, there were episodes of inattention and intermittent worsening of left‐sided hemiplegia, for which a magnetic resonance imaging (MRI) and continuous video electroencephalogram (vEEG) were conducted to rule out posterior reversible encephalopathy syndrome, seizure, and stroke. MRI brain noted an old right parietal cranioplasty and stable postsurgical encephalomalacia without recurrence of the tumor with mild generalized cerebral and cerebellar atrophy, chronic ischemic change in the pons, and benign choroid plexus xanthogranulomas. Figure 1.

**FIGURE 2:**
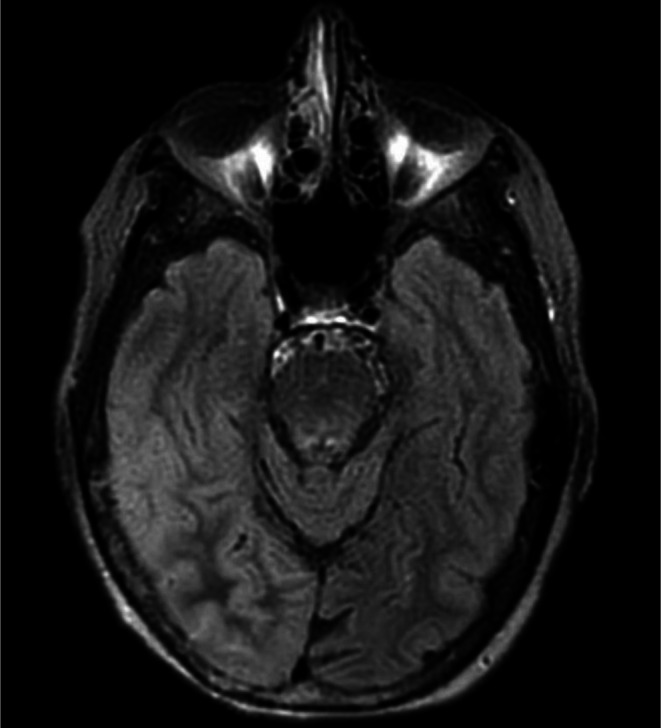
MRI brain (T2‐FLAIR) shows mild signal hyperintensity in the right parietal, occipital, and temporal regions compared to prior imaging, with sulcal effacement and mild mass effect.

**FIGURE 3:**
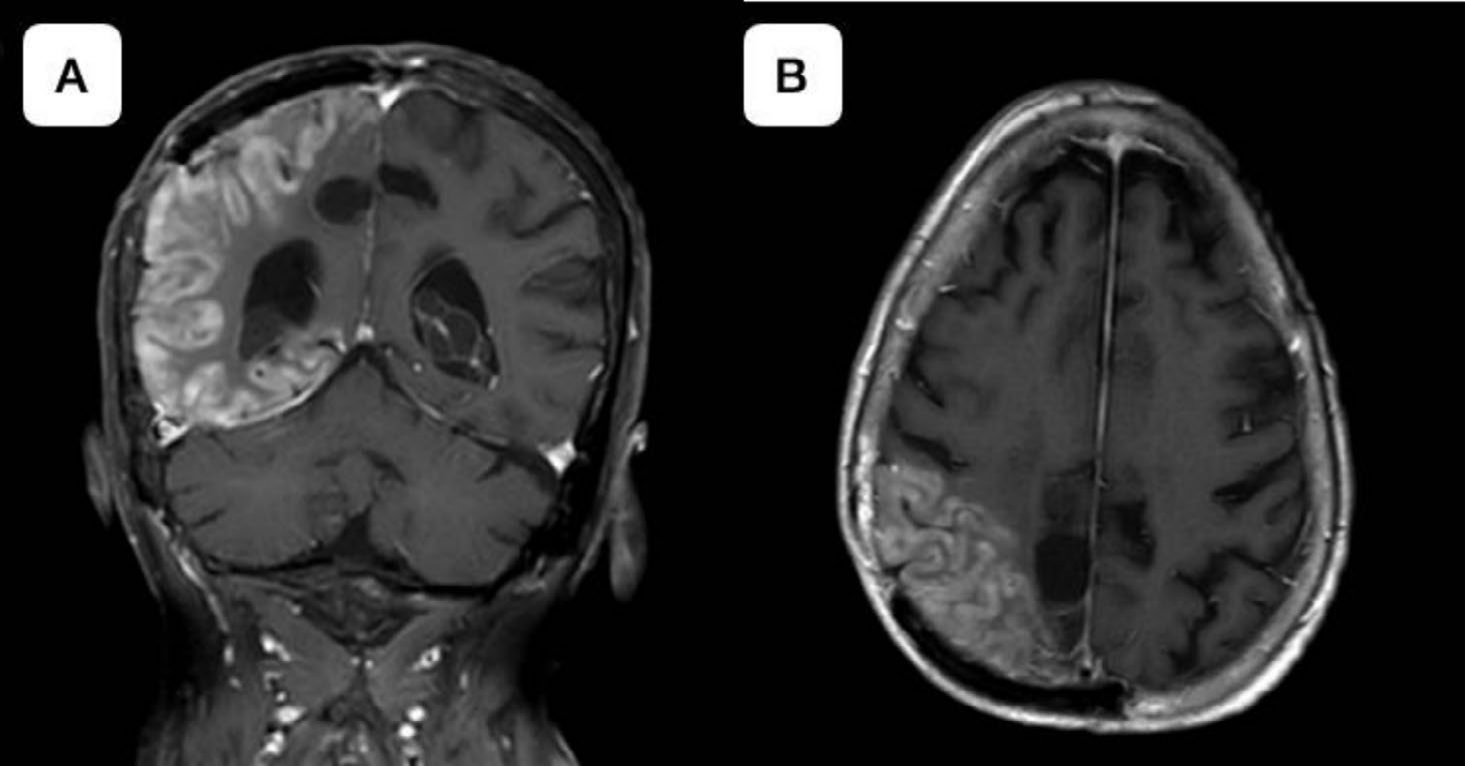
Contrasted MRI Brain. (A) Coronal view and (B) Axial view showing right parietal, temporal, occipital gyral enhancement with evidence of cortical swelling and mild diffusion restriction in DWI sequences in previously irradiated areas.

EEG revealed six focal onset seizures with impaired awareness originating in the right parietal‐occipital region. An initial loading dose of intravenous levetiracetam 1000 mg was administered, followed by 500 mg oral dosing every 12 h, which was later titrated to 750 mg every 12 h due to persistent seizure activity, with seven seizures in a 24‐h period. A repeat MRI conducted 4 days post‐admission, prompted by new‐onset seizures and migraine headache which contrasted from baseline headache history, demonstrated T2‐FLAIR (Fluid‐Attenuated Inversion Recovery) hyperintensity in the right parieto‐temporo‐occipital region Figure 2, along with sulcal effacement, cortical gyriform gadolinium enhancement and diffusion restriction, which was concerning for encephalitis or vasculitis Figure 3.

Cerebrospinal fluid (CSF) analysis was inconclusive, showing mild pleocytosis of 15 with a lymphocytic predominance and protein level of 52, while tests for meningoencephalitis, autoimmune encephalitis, Mitochondrial encephalomyopathy with lactic acidosis and stroke‐like episodes (MELAS) and paraneoplastic condition were negative. Infectious workup, including HIV, RPR, Lyme serology, and blood cultures, remained unremarkable. Empiric treatment for meningoencephalitis of unknown etiology was initiated with vancomycin, ceftriaxone, ampicillin, and acyclovir, and two doses of IVIG were administered without adequate response. MRI of the cervical, thoracic, and lumbar spine revealed chronic compression fractures at T10–L3 but no acute process, and brachial plexus MRI was unremarkable. The exclusion of differentials led to the diagnosis of stroke‐like migraine attacks after radiation therapy (SMART) syndrome.

### 2.3 Outcome and Follow‐Up

**FIGURE 4:**
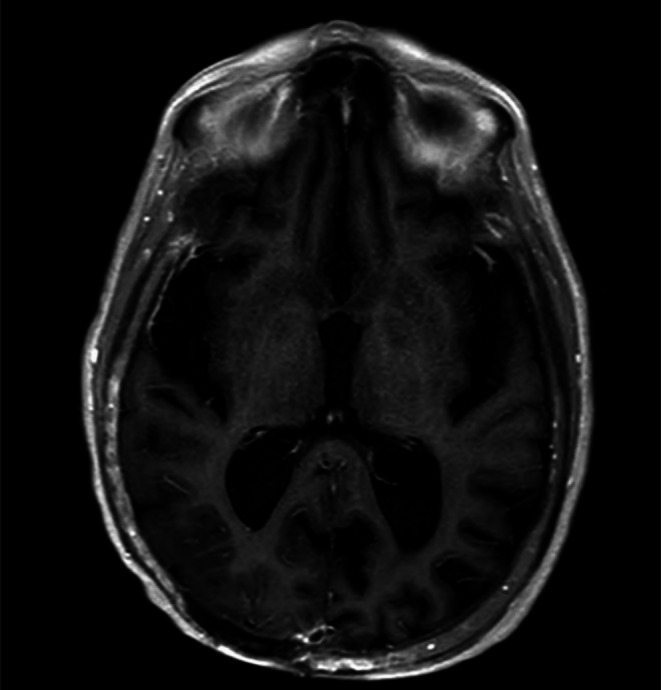
Contrasted MRI Brain showing resolution of cortical gyriform enhancement on repeat imaging.

She was stable for transfer to a tertiary care facility for further neurologic evaluation. Follow‐up MRI showed mild cortical edema and restricted diffusion in the right parietal and posterior temporal lobes, which was perhaps more consistent with postictal edema or subacute infarct but would be less likely tumor recurrence or postictal edema. The diffusion restriction resolved on a follow‐up MRI 2 weeks later Figure 4.

The patient tolerated antiepileptic (levetiracetam) and non‐steroidal anti‐inflammatory drugs (NSAID) for her headaches. She had a stable status during hospitalization with a normal level of consciousness and appropriate seizure and headache control. She was discharged with the addition of aspirin and verapamil.

## 3 Discussion

Cranial irradiations can have several deleterious effects on the neuronal tissue due to inflammation and disruption of the blood brain barrier. The radiation exposure results in endothelial damage through a downstream signaling that activates ceramide induced apoptosis [2]. Following endothelial damage, there is subsequent disruption of the blood brain barrier and radiation induced cerebrovascular effects [3]. A relatively rare and misdiagnosed complication of cranial irradiation is stroke‐like migraine attacks after radiation therapy (SMART) syndrome [4]. SMART syndrome is characterized by reversible neurological symptoms that last days to weeks. They often present as a triad of migraine‐type headaches, seizure, and stroke‐like focal deficit. It can occur as early as 2 years and can present even decades later [5]. Our case has a relatively longer latency period and presents approximately 35 years post radiation.

SMART syndrome has been documented in all age groups, including pediatric and adult populations. Although both sexes are affected equally, recent data suggest a higher recurrence risk in females [1, 6]. It has been reported following irradiation of a variety of primary intracranial malignancies, especially high‐grade gliomas such as astrocytoma in adults [7]. This presentation is similar to our case, who had a past history of astrocytoma.

It is noteworthy to differentiate preexisting migraine history in addition to the change in the headache pattern or association with other neurological deficits. The migraine headaches are often severe and unilateral with features of aura and are experienced by almost two‐thirds of patients. A majority of patients also have focal seizures that precede or accompany other neurological deficits. The development of subacute stroke has also been frequently implicated which can persist for days or weeks before improving [8]. The common findings include visual disturbances like homonymous hemianopsia or visuospatial neglect, sensory deficits, unilateral limb weakness, facial palsy and language impairment. Unlike an acute ischemic stroke, SMART syndrome deficits evolve slower and are reversible within one to 3 months [8, 9].

Given the overlap with stroke and tumor recurrence, a thorough radiological evaluation is the cornerstone of diagnosis. Magnetic resonance imaging (MRI) brain in the acute phase shows unilateral cortical T2‐FLAIR (Fluid‐Attenuated Inversion Recovery) hyperintensity with gyriform enhancements which corresponds to the region of previous high‐dose irradiation. The temporal and parietal lobes followed by occipital and frontal lobes are most commonly involved as these sites receive significant radiation. The perfusion imaging with magnetic resonance or computed tomography (CT) shows transient hyperperfusion due to loss of autoregulation or seizure‐related increased blood flow which normalizes or reduces on follow‐up [1]. The transient nature of enhancement and lack of mass effect are cues that favor SMART syndrome over tumor recurrence. The vascular territory distribution and diffusion restriction in diffusion weighted imaging (DWI) can distinguish ischemic strokes from SMART syndrome. Cerebrospinal fluid (CSF) findings are generally unremarkable in SMART syndrome but can be useful to rule out autoimmune encephalitis. Electroencephalography (EEG) captures focal epileptiform discharges or even subclinical status epilepticus, which can necessitate aggressive seizure management.

There is no formal evidence‐based treatment guidelines for SMART syndrome due to its rarity and lack of clinical trials. The management is largely focused on treating acute symptoms, preventing complications, and monitoring for improvement or recurrence. Patients are often hospitalized for stroke‐like presentation, and supportive measures include hydration, pain control, and close neurological observation with physical examination and repeat imaging. Seizure control with appropriate anti‐seizure medication should be initiated promptly. The headache management involves using non‐steroidal anti‐inflammatory drugs and anti‐emetics for nausea. Triptans and ergotamines should be avoided, especially in stroke‐mimic suspected scenarios. High‐dose steroids such as dexamethasone and methylprednisolone have been empirically used in SMART syndrome to reduce cerebral edema and inflammation in the affected areas [1, 8]. Antiplatelet therapy (aspirin) and calcium channel blockers (verapamil) can help in reducing the recurrence and severity of SMART syndrome [4, 10]. Ketamine has shown useful in the case of refractory seizures in SMART syndrome [10]. Close follow‐up is essential after an acute SMART episode, especially in older adults and females who have a higher possibility of incomplete recovery and increased risk of recurrence [11].

In conclusion, this case highlights the importance of recognizing SMART syndrome as a potential late complication of cranial irradiation, especially due to its prolonged latency. It is critical to identify SMART earlier through characteristic imaging findings and exclusion of alternative etiologies to avoid unnecessary interventions.

## Author Contributions

**Shiv Jha:** resources, visualization, writing – review and editing. **Yugant Khand:** conceptualization, writing – original draft, writing – review and editing. **Surabhi Ranjan:** supervision, writing – review and editing. **Monika Pandit:** writing – review and editing. **Eric Mariuma:** writing – review and editing.

## Funding

The authors have nothing to report.

## Ethics Statement

The authors have nothing to report.

## Consent

Written informed consent was obtained from the patient for publication of clinical details and accompanying images.

## Conflicts of Interest

The authors declare no conflicts of interest.

## Data Availability

Data available on request from the authors.
